# Musculoskeletal Ultrasound Diagnosis of Symptomatic Hoffa’s Fat Pad Ganglion Cyst Successfully Managed Nonoperatively: A Case Report

**DOI:** 10.7759/cureus.18665

**Published:** 2021-10-11

**Authors:** Laurie A Ramrattan, Ghania Masri, Gurjit S Kaeley

**Affiliations:** 1 Department of Medicine/Rheumatology, University of Florida College of Medicine – Jacksonville, Jacksonville, USA; 2 Department of Medicine, University of Florida College of Medicine – Jacksonville, Jacksonville, USA

**Keywords:** knee injection, knee joint, hoffa’s fat pad, ganglion cyst, musculoskeletal ultrasonography

## Abstract

Ganglion cysts (GCs) can arise from a variety of different areas, but those arising from Hoffa’s fat pad in the knee are relatively rare. A number of different types of cysts are also found in the knee, including meniscus cysts, proximal tibiofibular cysts, and cruciate ligament ganglion cysts. In this case report, a 54-year-old female presented with left knee pain and swelling for eight weeks. In-office musculoskeletal ultrasound (US) was used to diagnose a Hoffa’s fat pad ganglion cyst and aid in targeted steroid injection. The patient was followed up 21 weeks after the injection. She was pain-free, and US findings showed the cyst had significantly decreased in size. This case shows how musculoskeletal ultrasound, a relatively inexpensive diagnostic modality, can be used to accurately diagnose the cause of knee pain, guide an in-office procedure for the treatment of Hoffa's fat pad ganglion cyst, and aid in monitoring.

## Introduction

A ganglion cyst (GC) is a cystic, tumorlike lesion demarcated by dense connective tissue and filled with gelatinous fluid rich in hyaluronic acid and other mucopolysaccharides and is of unknown origin [1]. A GC arising from Hoffa’s fat pad is relatively rare, and the incidence on magnetic resonance imaging (MRI) is 1% [2], which correlates with the incidence reported on arthroscopy of 0.8%-1.1% [3]. The infrapatellar fat pad, also known as Hoffa’s fat pad, is intracapsular but extrasynovial. A variety of abnormal conditions can occur in Hoffa’s fat pad, including GC [4]. Thus, a Hoffa’s fat pad GC causing knee pain is uncommon, and surgical excision with open ganglionectomy is curative [5] and is the standard of care. Musculoskeletal ultrasound (US) can not only visualize Hoffa’s fat pad cysts but also allow guided aspiration and injection [6,7]. Ultrasound is especially useful when cysts are smaller, deeper, and located near vessels and nerves [7]. Data on GC's ultrasound-guided treatment is available primarily for the upper extremities [8,9].

## Case presentation

**Figure 1:**
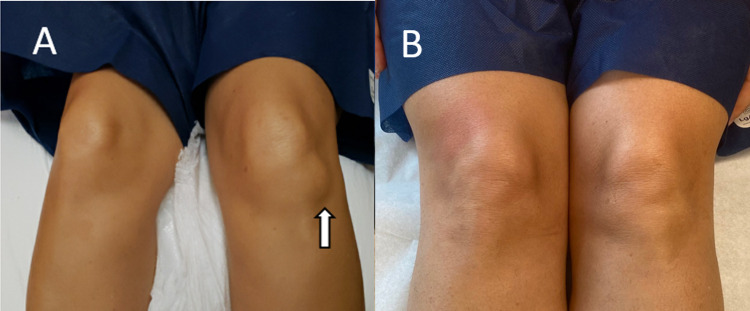
A: Preinjection knee showing ganglion cyst (arrow). B: Postinjection knee (21 weeks after injection)

A 54-year-old female presented with left knee pain for eight weeks and swelling on the knee's anterolateral aspect. There was a remote history of falling on her knees from her bicycle 16 weeks before the presentation. The pain was sharp, worse with walking resulting in limping, limiting the patient’s ability to walk, and more severe at night. No constitutional symptoms were reported. The pain gradually worsens and was severe enough to interfere with her regular exercise and sleeping. Over-the-counter topical analgesics, therapeutic doses of NSAID, and bracing were tried without improvement. On examination, there was a 5 x 5 cm tender lump on the anterior-lateral aspect of the left knee (Figure 1A). The swelling was firm in consistency, tender to palpation, and mobile. Knee range of motion was limited secondary to pain. The opposite knee was normal. The plain radiographs were normal.

**Figure 2:**
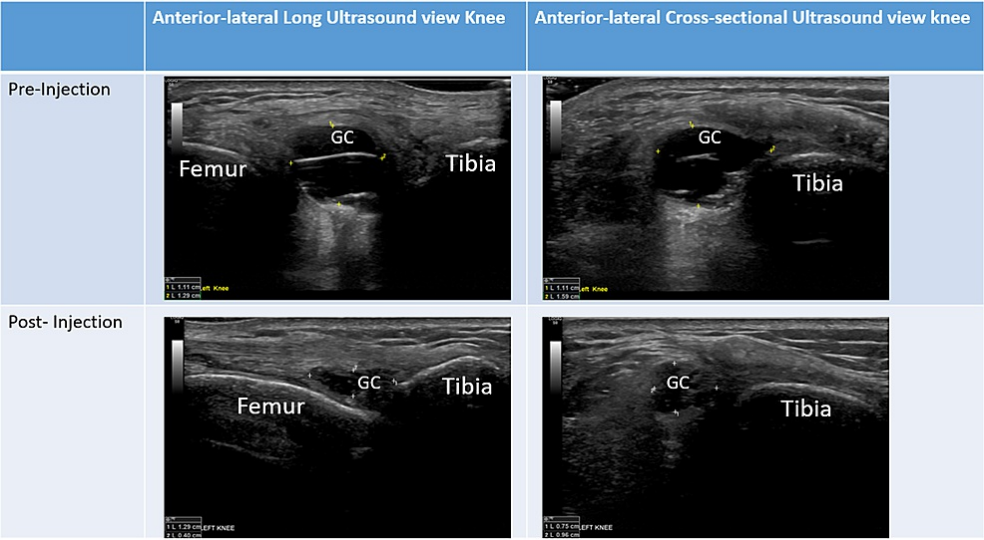
Lateral infrapatellar views acquired with the knee placed at 30° flexion. Left panels: Lateral long axis (sagittal plane) scans with the probe place proximally on the femur and distally on the tibial. Top left preinjection panel: An anechoic ganglion cyst (GC) that has posterior acoustic enhancement over Hoffa’s fat pad as well as septations within the cyst. Top right panel: Orthogonal views in the transverse plane demonstrating the ganglion cyst adjacent to the tibia with similar ultrasound characteristics. Bottom two panels: Postinjection images acquired in the same location and demonstrating reduction in the size of the ganglion cyst GC: ganglion cyst

Musculoskeletal ultrasound of the lateral Hoffa’s fat pad revealed a 1.11 x 1.29 cm GC on longitudinal view and a 1.11 x 1.59 cm CG on the cross-sectional view of the anterior-lateral knee (Figure 2). The GC had anechoic regions with septations, and posterior acoustic enhancement was seen. There was no connection to the joint space, and probe palpation of this region reproduced the patient's symptoms. Doppler signal was not increased. Mild to moderate fluid was seen in the deep infrapatellar bursa. The ganglion cyst was accessed under sonographic visualization using a 21G 1.5-inch needle and 2% lidocaine for needle track and soft tissue anesthesia. Despite repositioning the needle in multiple directions, no material could be aspirated. Under sonographic visualization, 40 mg methylprednisolone was injected into the cyst without any complications.

Over the next week after the methylprednisolone injection of Hoffa’s fat pad ganglion cyst, the patient’s knee pain and swelling improved. She was pain-free two weeks after the injection, and her walking returned to normal. At 21 weeks postinjection, the patient remains pain-free without reoccurrence of the knee swelling (Figure 1B). A repeat ultrasound was also performed at this time, which showed that the cyst was significantly smaller and measured 1.29 x 0.40 cm on longitudinal view and 0.75 x 0.96 cm (Figure 2) on cross-sectional view.

## Discussion

Although MRI has been used for the diagnosis of GC and is the best imaging modality for cysts around the knee joint [5], the use of US has grown in popularity. Sonography is a relatively inexpensive, convenient, in-office procedure that can be used for both diagnostic and therapeutic purposes. The characteristic ultrasound features of a GC are a well-defined, anechoic, or mildly complex soft tissue lesion with posterior acoustic enhancement, lacking internal vascularity and having variable degrees of compressibility [7]. Compared with a synovial cyst, minimal displacement of fluid occurs in a ganglion cyst upon probe pressure or palpation [10]. Ganglion cysts contain highly viscous fluid, which explains why it cannot be aspirated easily [5] and is difficult to compress or demonstrate fluid displacement.

Ju et al. (2017) studied the outcome of ultrasound-guided therapy of knee and foot ganglion cysts, but they did not specifically mention treating a Hoffa’s fat pad ganglion cyst. The majority of literature reviewed reported surgical excision of ganglion cyst as treatment [3,5]. However, surgery can have postoperative morbidity and should not be the first treatment option. Mithilesh et al. (2019) studied reduction in the volume of ganglion cysts involving the wrist and ankle regions after steroid injection. The participants had US volume measurements of their cyst and guided injection, and six months later, they were reaccessed with US volume measurements. There was a reduction in the size of more than half in 45% of participants, with 10% being completely resolved [11]. They, however, did not differentiate between the types of cysts, did not include knee cysts, and did not mention if the pain and disability caused by the cysts were improved. Also, the reduction in the size of the cyst can be due to the natural cause of the disease. Dias et al. (2007) showed that resolution occurred in 58% of patients over a 70-month period [12]. In our case, a single injection of 40 mg methylprednisolone not only resulted in symptomatic relief but also led to a substantial reduction in the size of Hoffa’s fat pad ganglion cyst.

## Conclusions

This case illustrates that a US-guided local steroid injection may provide sufficient relief to a patient with a Hoffa’s fat pad GC and has the potential of avoiding excisional surgery. Steroid injection decreased the size of the cyst significantly and hence improved the patient’s symptoms. This case also demonstrates the importance of using sonography at the point of care, which led to prompt diagnosis and therapy.

## References

[1] Beaman FD, Peterson JJ (2007). MR imaging of cysts, ganglia, and bursae about the knee. Magn Reson Imaging Clin N Am.

[2] Bui-Mansfield LT, Youngberg RA (1997). Intraarticular ganglia of the knee: prevalence, presentation, etiology, and management. AJR Am J Roentgenol.

[3] Krudwig WK, Schulte KK, Heinemann C (2004). Intra-articular ganglion cysts of the knee joint: a report of 85 cases and review of the literature. Knee Surg Sports Traumatol Arthrosc.

[4] Vaishya R, Irfan S (2012). Arthroscopic resection of localized pigmented villonodular synovitis of the knee. Apollo medicine.

[5] Vaishya R, Kansagra A, Agarwal AK, Vijay V (2020). A giant ganglion cyst arising from lateral Hoffa's fat pad of the knee. J Orthop Case Rep.

[6] Jose J, Silverman E, Kaplan L (2011). Symptomatic ganglion cyst of the popliteus tendon treated with ultrasound-guided aspiration and steroid injection: a case report. Sports Health.

[7] Ju BL, Weber KL, Khoury V (2017). Ultrasound-guided therapy for knee and foot ganglion cysts. J Foot Ankle Surg.

[8] Bianchi S, Abdelwahab IF, Zwass A, Giacomello P (1994). Ultrasonographic evaluation of wrist ganglia. Skeletal Radiol.

[9] Breidahl WH, Adler RS (1996). Ultrasound-guided injection of ganglia with coricosteroids. Skeletal Radiol.

[10] Cardinal E, Buckwalter KA, Braunstein EM, Mih AD (1994). Occult dorsal carpal ganglion: comparison of US and MR imaging. Radiology.

[11] Sinha MK, Mishra P, Mishra TS, Barman A (2019). Aspiration and steroid injection in ganglion cysts: an ultrasound guided evaluation of the response. J Clin Orthop Trauma.

[12] Dias JJ, Dhukaram V, Kumar P (2007). The natural history of untreated dorsal wrist ganglia and patient reported outcome 6 years after intervention. J Hand Surg Eur Vol.

